# Characterization of natural variation in North American Atlantic Salmon populations (Salmonidae: *Salmo salar*) at a locus with a major effect on sea age

**DOI:** 10.1002/ece3.3132

**Published:** 2017-06-17

**Authors:** Henrik Kusche, Guillaume Côté, Cécilia Hernandez, Eric Normandeau, Damien Boivin‐Delisle, Louis Bernatchez

**Affiliations:** ^1^ Département de Biologie Institut de Biologie Intégrative et des Systèmes (IBIS) Université Laval Québec QC Canada; ^2^ Present address: Thünen Institute of Fisheries Ecology Hamburg Germany

**Keywords:** dual‐index barcoding, genes of large effect, Illumina^®^ MiSeq, life history, paired‐end amplicon sequencing, single nucleotide polymorphisms

## Abstract

Age at maturity is a key life‐history trait of most organisms. In anadromous salmonid fishes such as Atlantic Salmon (*Salmo salar*), age at sexual maturity is associated with sea age, the number of years spent at sea before the spawning migration. For the first time, we investigated the presence of two nonsynonymous *vgll3* polymorphisms in North American Atlantic Salmon populations that relate to sea age in European salmon and quantified the natural variation at these and two additional candidate SNPs from two other genes. A targeted resequencing assay was developed and 1,505 returning adult individuals of size‐inferred sea age and sex from four populations were genotyped. Across three of four populations sampled in Québec, Canada, the late‐maturing component (MSW) of the population of a given sex exhibited higher proportions of SNP genotypes 54Thr_*vgll3*_ and 323Lys_*vgll3*_ compared to early‐maturing fish (1SW), for example, 85% versus 53% of females from Trinité River carried 323Lys_*vgll3*_ (*n*_MSW_ = 205 vs. *n*_1SW_ = 30; *p < *.001). However, the association between *vgll3* polymorphism and sea age was more pronounced in females than in males in the rivers we studied. Logistic regression analysis of *vgll3* SNP genotypes revealed increased probabilities of exhibiting higher sea age for 54Thr_*vgll3*_ and 323Lys_*vgll3*_ genotypes compared to alternative genotypes, depending on population and sex. Moreover, individuals carrying the heterozygous *vgll3* SNP genotypes were more likely (>66%) to be female. In summary, two nonsynonymous *vgll3* polymorphisms were confirmed in North American populations of Atlantic Salmon and our results suggest that variation at those loci correlates with sea age and sex. Our results also suggest that this correlation varies among populations. Future work would benefit from a more balanced sampling and from adding data on juvenile riverine life stages to contrast our data.

## 1 INTRODUCTION

One of the largest contemporary challenges is to balance the availability of biological resources with an increasing demand for exploitation through a consistently growing world population (Ludwig, Hilborn, & Walters, 1993). Forestry, hunting, and fisheries are classical ways to exploit biological resources. However, those resources are naturally limited in their abundance and many species are vulnerable to overexploitation and disturbance. The issue of exploitation and habitat disturbance of fisheries resources increased dramatically over the last decades (Christensen et al., 2003; Limburg & Waldman, 2009; Pauly et al., 2002). Harvesting fish from natural populations reduces genetic, morphological, and life‐history diversity (Allendorf, England, Luikart, Ritchie, & Ryman, 2008; Conover & Munch, 2002; Kuparinen & Merilä, 2007). Consequently, a thorough understanding of key biological traits in exploited fish populations, that is, major traits characteristic of an individual's fitness, life history, or age, can be crucial for the persistence and management of this valuable aquatic resource.

Fish stock biodiversity has been traditionally monitored by assessing general abundance, size at age, morphological and life‐history variation, etc. (Hilborn & Walters, 1992; Pauly et al., 2002). In addition to phenotypic approaches, putatively neutral genetic markers serve to delineate population structure and diversification in numerous cases (Cuéllar‐Pinzón, Presa, Hawkins, & Pita, 2016; Hauser & Seeb, 2008). Genomic approaches are currently revolutionizing our understanding of population structuring and diversification processes. Next‐generation sequencing technologies increasingly contribute to resolve fundamental and applied aspects in fisheries and aquaculture. For example, SNP‐based approaches (single nucleotide polymorphisms) allow researchers to identify genes and genomic regions associated with major phenotypic traits, to discriminate species and fish stocks, or to trace fish and fisheries products along the supply chain (Bernatchez, 2016; Cuéllar‐Pinzón et al., 2016; Elmer, 2016; Hemmer‐Hansen, Therkildsen, & Pujolar, 2014; Valenzuela‐Quiñonez, 2016). Molecular genetic approaches open up unprecedented avenues to manage and to restore natural variation in exploited species, in particular if genetic markers associate with key biological traits (Allendorf et al., 2008).

The Atlantic Salmon (*Salmo salar*) is an exploited migratory fish species of immense economic and cultural importance for the nations abutting the North Atlantic. However, throughout their native range in the Northern Hemisphere, the species is in serious peril. The International Council for the Exploration of the Sea has documented a dramatic decline of Atlantic Salmon over the last decades (Chaput, 2012; ICES 2013). Various factors likely contributed to this reduction in overall abundance, including numerous anthropogenic impacts (Limburg & Waldman, 2009; Otero et al., 2011) such as excessive overexploitation, not only through offshore fisheries at the open sea, but also in riverside areas, where the fish return from their sea migration to spawn. For instance, since the 1970s, the marine mortality of Atlantic Salmon in European populations has increased from 70% to over 90% in 2005 (Friedland et al., 2009). The WWF estimates that of 2,005 traditionally salmon‐bearing river populations, more than 40% are threatened and 15% have gone extinct already (World Wildlife Fund 2001). Many of the remaining populations exhibit substantially reduced genetic and phenotypic diversity (King et al., 2007). Previous work documented the evolutionary consequences of extensive size‐selective harvesting that frequently manifests in alteration of certain life‐history traits in the population such as reduced growth rates, overall smaller body size, and an earlier onset of sexual maturation (Jorgensen et al., 2007; Kuparinen & Merilä, 2007; Quinn, McGinnity, & Cross, 2006). For example, in many wild populations, the age at which individuals return from their marine migration to spawn decreased significantly in the last decades (Friedland et al., 2009; Otero et al., 2011). The life cycle starts with spawning in freshwater rivers. Various juvenile phases (alevin → fry → parr → smolt) can be found in riverine nursery grounds. After several years in the riverine habitat, the smolts finally migrate to their feeding grounds at sea. Atlantic Salmon spend either one winter (1SW) or multiple winters (MSW; up to five recorded) at sea before they mature and return to their river of origin to spawn in order to complete their life cycle. Repeated spawning migrations in subsequent years (iteroparity) occur at low frequencies of ca. 11% but vary among populations (Fleming, 1998). The number of winters at sea before maturation is termed sea age at maturity, hereinafter “sea age.”

Sea age has been considered as crucial for sustainable population persistence over multiple generations in another salmonid, the Sockeye Salmon (*Oncorhynchus nerka*) (Schindler et al., 2010), and a broad distribution of sea age in a population is a reliable predictor of genetic diversity in Atlantic Salmon (Vähä, Erkinaro, Niemelä, & Primmer, 2007). Decreasing sea age also reduces the value of populations as it is the larger multi‐sea winter fish that anglers are attracted to. In summary, sea age defines population diversity and structuring to a large extent and has a great potential to serve as reference trait for biodiversity assessments and for the management of exploited Atlantic Salmon populations.

Recently, variation in sea age in Atlantic Salmon has been attributed to a major selective sweep containing the candidate locus (*vgll3*,* vestigial‐like family member 3,* a 4‐kb gene located on chromosome 25 consisting of 4 exons of 460, 440, 570, and 600 bp and no splicing variants) in European populations (Ayllon et al., 2015; Barson et al., 2015). A combined approach of a SNP‐based genomewide association study on 1,404 individuals from 57 populations and whole‐genome resequencing of 32 individuals revealed that *vgll3* explained 39% of the variation in sea age (Barson et al., 2015). Moreover, an independently conducted study on both domesticated and wild Atlantic Salmon from Western Norway narrowed down the candidate region to a 2.4‐kb stretch within the *vgll3* gene (Ayllon et al., 2015). However, the causative SNPs or other possible causal variants still remain to be elucidated.

The two alleles at the *vgll3* locus are associated either with early (E) maturation and thus low sea age, or with late (L) maturation and higher sea age (Ayllon et al., 2015; Barson et al., 2015). Moreover, sex‐dependent dominance of E and L alleles was documented (Barson et al., 2015). The E allele promoting earlier maturation is most abundant in males (EE most frequent, EL moderate, LL most rare), whereas in females the L allele promoting delayed maturation seems to be selected for (EE most rare, EL moderate, LL most frequent) (Barson et al., 2015). Interestingly, *vgll3* is also associated with the onset of puberty and adiposity in humans (Cousminer et al., 2013), suggesting that this gene might be functionally highly conserved across vertebrates. Because late‐maturing fish with larger body mass (higher sea age) exhibit higher reproductive success and females mature later than males on average (Fleming & Einum, 2010), sea age is a trait of great interest to understand sexual conflict, that is, sex‐differential selection on a trait with a common genetic basis.

Two strongly linked nonsynonymous SNPs were independently identified within *vgll3* (Met54Thr_*vgll3*_ and Asn323Lys_*vgll3*_) (Ayllon et al., 2015; Barson et al., 2015) and were predicted to alter protein function and structure (Barson et al., 2015). 1SW males predominantly exhibited methionine and asparagine at amino acid positions #54 and #323 in the *vgll3* protein, respectively, whereas 3SW males more likely exhibited threonine and lysine at those positions and Met54Thr_*vgll3*_ and Asn323Lys_*vgll3*_ explained 33% and 36% of sea age (Ayllon et al., 2015). Besides *vgll3*, two additional, but less supported candidate genes for sea age were identified: *akap11* (*a‐kinase anchor protein 11*) (Ayllon et al., 2015; Barson et al., 2015) and *six6* (Barson et al., 2015). *Akap11* is located on the same chromosome (chm25) as *vgll3* and within the main selective sweep associated with sea age (Ayllon et al., 2015; Barson et al., 2015), whereas *six6* is situated in a region on chromosome 9 and in strong linkage disequilibrium with multiple other genes (Barson et al., 2015). In humans, the expression of *akap11* correlates with spermatogenesis (Vijayaraghavan et al., 1999), whereas *six6,* a transcription factor involved in the hypothalamic–pituitary–ovarian axis, is associated with pubertal height, growth, and age at maturity (Perry et al., 2014). A nonsynonymous mutation was characterized in *akap11* which was predicted to change protein structure (Val214Met_*akap11*_) (Barson et al., 2015). 1SW fish exhibited predominantly the Val214 variant of *akap11*, whereas the MSW fish carried the Met214 variant (Ayllon et al., 2015). An additional SNP marker (termed *SIX6*_TOP_ (Barson et al., 2015) due to its proximity to the *six6* candidate gene) was associated with sea age in the pooled sample of 57 populations, but not after correction for population stratification. *SIX6*_TOP_ explained 5% and 3% of the size variation within sea age categories in females and males (Barson et al., 2015).

In this study, we assessed for the first time in North American Atlantic Salmon populations the natural variation at the previously identified loci associated with sea age. The nonsynonymous single nucleotide variants in candidate genes *vgll3* and *akap11* were of particular interest because of their potential to alter protein function and structure and thus of their putative biological significance for sea age. We developed targeted resequencing assays for the nonsynonymous mutations Met54Thr_*vgll3*_, Asn323Lys_*vgll3*_, and Val214Met_*akap11*_, as well as for *SIX6*_TOP,_ and related genetic and phenotypic variation at the individual level in four Canadian rivers that varied in their 1SW to MSW ratios. The rationale was to find out whether these candidate SNPs existed in North American populations and how they associate with sea age.

## 2 MATERIALS AND METHODS

### 2.1 Sample collection and selection of data set

**Figure 1:**
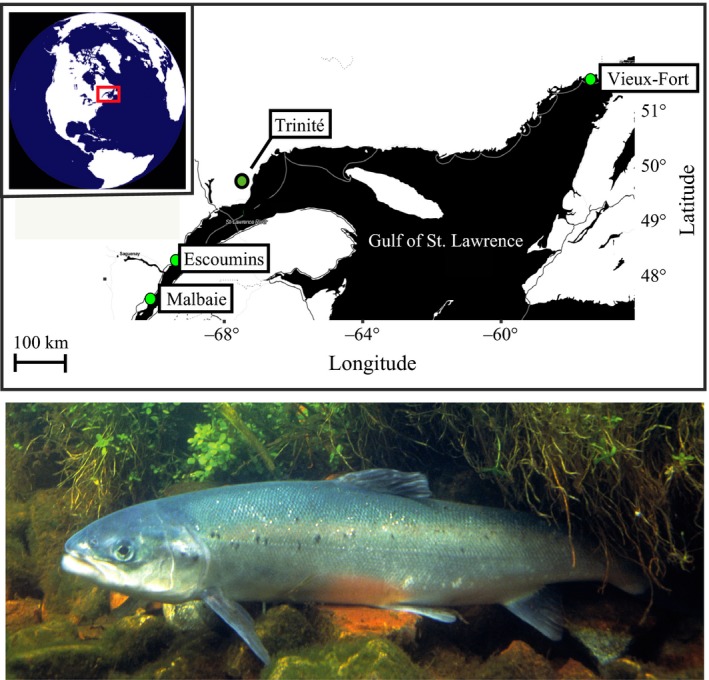
Atlantic Salmon and map of sampling locations in the Québec province (Canada). Specimen photograph by Hartley, William W.—U.S. Fish & Wildlife Service. Licensed under public domain via Wikimedia Commons—http://commons.wikimedia.org

Returning adult Atlantic Salmon were collected between 2003 and 2015 from rivers Malbaie, Escoumins, Trinité, and Vieux‐Fort in the Canadian Québec province (Figure 1). Sampling was done under direction of the Ministry of Forest, Wildlife and Parks of Québec (MFFP) in the framework of annual monitoring censuses and previous studies (Milot, Perrier, Papillon, Dodson, & Bernatchez, 2013; Richard, Dionne, Wang, & Bernatchez, 2013). These rivers harbor different average levels of sea age at maturity. Malbaie and Escoumins rivers have relatively lower proportions of 1SW fish (23% and 38% of the returning adult individuals), whereas on average 63% and 90% 1SW fish are found in rivers Trinité and Vieux‐Fort over five recent consecutive years, respectively (Cauchon, 2015). Fish were caught with seine nets, and fork length was measured to the nearest cm in the field. A small piece of fin tissue was conserved in 97% ethanol for later DNA extraction in the laboratory.

1SW and MSW assignment was based on fork length, the distance between the tip of the snout to the most distal point of the caudal fin rays (Fig. S1). A fork length of 63 cm is an established empirical threshold to discriminate between 1SW and MSW fish in Québec (MFFP 2016). Individuals measuring 63 ± 5 cm were excluded from the study to further reduce ambiguity in the assignments, that is, individuals <58 cm were assigned to the 1SW group and those >68 cm were assigned to the MSW group. Sample sizes were limited to an *n* of 50 for 1SW fish per sampling year and river to an *n *=* *100 for MSW fish (Table S1), such that in total 1,505 individuals (653 1SW and 852 MSW) were included. To maximize size variation, individuals representing a broad range of fork length between 36 and 110 cm were selected. Scales from 800 individuals were collected by MFFP staff and used to infer life history from the spacing of the scale circuli. A total of 403 fish were determined as 1SW and 397 fish were determined as 2SW. All 397 2SW fish (scales) were also classified by fork length as MSW, whereas 394 of the 403 1SW fish (scales) were also classified by fork length as 1SW. Multiple spawners, as inferred from scale readings, were entirely excluded, but it is still conceivable that a small number of multiple spawners and >3SW fish remain in the MSW component that had no available scale reads.

### 2.2 DNA extraction and normalization

DNA was extracted from fin clips using a salt extraction method (Aljanabi & Martinez, 1997). Twenty‐one negative controls were used as blanks and quality control during the library preparation. Quality and quantity of extracted DNA were measured in 96‐well plate format using a Spark 10M multimode microplate reader (Tecan) (4 μl DNA extract and 96 μl ddH_2_O). DNA concentrations were normalized using postmeasurement dilutions to reach working concentrations of 10–25 ng/μl for each sample.

### 2.3 Sex determination

All individuals were sexed using polymerase chain reaction (PCR) (Quéméré et al., 2014; Yano et al., 2013). The male‐specific *sdY* gene was targeted, and therefore during agarose gel electrophoresis, only male individuals and the positive control showed a fluorescent band. The PCR cocktail was 2 μl DNA extract, 2 μl of 5 × Colorless GoTaq^®^ Flex Buffer, 0.65 μl of 25 mmol/L MgCl_2_, 0.8 μl of 10 mmol/L dNTPs, 0.5 μl of 10 μmol/L forward primer (5′GGGCCTATGAATTTCTGATG), 0.5 μl of 10 μmol/L reverse primer (ACAGATTTGCGACATGAACA), 1.85 μl of ddH_2_O, and 0.2 μl GoTaq^®^ Flexi DNA polymerase. PCRs were conducted in Biometra^®^ thermocyclers as follows: 95°C—2 min, 35 cycles [95°C—45 s, 60°C—45 s, 72°C—45 s], 60°C—30 min, 4°C—∞.

### 2.4 Primer design and amplicon library preparation

DNA amplifications were performed using newly designed gene‐specific primers (this study) in a two‐step dual‐indexed PCR approach specifically designed for Illumina instruments by the Plateforme d'Analyses Génomiques (IBIS, Université Laval, Québec city, Canada). Please note that primers used in this work contain Illumina‐specific sequences protected by intellectual property (Oligonucleotide sequences © 2007–2013 Illumina, Inc. All rights reserved. Derivative works created by Illumina customers are authorized for use with Illumina instruments and products only. All other uses are strictly prohibited.). Library preparation followed a two‐step PCR design, with PCR1 targeting the candidate region and attaching the Illumina adaptors and PCR2 adding traceable barcodes for each individual to the amplicons. The PCR1 primers targeting the SNPs causing nonsynonymous amino acid changes in the *vgll3* protein (Primer set “VGLL3_54” targeting Met54Thr_*vgll3*_ and “VGLL3_323” targeting Asn323Lys_*vgll3*_) and the *akap11* protein (“AKAP11” targeting Val214Met_*akap11*)_, as well as for the *SIX6*_TOP_ SNP (“SIX6”), were designed using Primer3 (Koressaar & Remm, 2007; Untergasser et al., 2012) based on the information provided in recently published manuscripts (Ayllon et al., 2015; Barson et al., 2015) and the nucleotide and amino acid sequences for *vgll3*,* akap11,* and *six6* of the Atlantic Salmon reference genome deposited at GenBank (GCA_000233375.4) (Lien et al., 2016) (Table S2). Target lengths ranged between 406 and 444 bp. PCR1 primers were designed with similar annealing temperatures to ensure multiplex PCR, as well as with attached Illumina^®^ adaptors (5′‐sequence added to forward primer: ACACTCTTTCCCTACACGACGCTCTTCCGATCT, 5′‐sequence added to reverse primer: GTGACTGGAGTTCAGACGTGTGCTCTTCCGATCT). PCR1 primers were diluted to 10 μmol/L and were tested in single and multiplex PCRs. Amplification of the PCR1 constructs was verified using gel electrophoresis. Given the similarity in fragment lengths, multiplex PCR co‐amplification was confirmed using Agilent 2100 Bioanalyzer electrophoresis chips and a M13 fluorescent labeling approach (Schuelke, 2000). Co‐amplification in a four‐fragment multiplex PCR1 could not reliably be confirmed with either method, so for the library preparation, two separate duplex PCR1 were pooled. The first duplex PCR1 targeted the amplicons VGLL3_323 and VGLL3_54 and the second duplex PCR1 included AKAP11 and SIX6. PCR cocktails and conditions for both duplex PCR1 were identical, apart from the reaction‐specific primer sets: 2 μl DNA extract, 2 μl of 5 × Colorless GoTaq^®^ Flex Buffer, 0.65 μl of 25 mmol/L MgCl_2_, 0.8 μl of 10 mmol/L dNTPs, 2 * 0.4 μl of 10 μmol/L forward primer, 2 * 0.4 μl of 10 μmol/L reverse primer, 2.75 μl of ddH_2_O, and 0.2 μl GoTaq^®^ Flexi DNA polymerase. PCR1 was conducted in Biometra^®^ thermocyclers: 95°C—2 min, 33 cycles [95°C—45 s, 56°C—45 s, 72°C—45 s], 72°C—5 min, 4°C—∞. Following amplification, 2.5 μl of each duplex PCR1 product was pooled in a fresh 96‐well plate, and a subset of 5 μl of pooled PCR1 product was enzymatically cleaned with 1 μl of ExoSAP‐IT^®^ in a thermocycler (37°C—15 min, 80°C—15 min, 4°C—hold). The cleaned and pooled PCR1 product was diluted with ddH_2_0 to the ratio of 1:10.

In order to trace each of the 1,505 individuals in downstream bioinformatic analyses, the PCR1 amplicons were dual‐index barcoded using a second PCR (PCR2), which for each of the 1,505 individuals added unique combinations of barcode adaptors to the PCR1 amplicons that bind to the Illumina^®^ adaptors at the 5′‐ and 3′‐end of each PCR1 amplicon. The generic PCR2 forward primer was AATGATACGGCGACCACCGAGATCTACAC [index1] ACACTCTTTCCCTACACGAC and the generic reverse PCR2 primer was CAAGCAGAAGACGGCATACGAGAT [index2] GTGACTGGAGTTCAGACGTGT. The individual‐specific barcode sequence mix was provided by the IBIS (Institut de Biologie Intégrative et des Systèmes, University Laval) genomic center staff in the format of 16 ready‐to‐use 96‐well plates, thus allowing discriminating a maximum of 1,536 individuals.

The PCR2 cocktail contained 2 μl of 1:10 diluted ExoSAP cleaned PCR1 product, 2 μl of 5 × Colorless GoTaq^®^ Flex Buffer, 0.65 μl of 25 mmol/L MgCl_2_, 0.8 μl of 10 mmol/L dNTPs, 1 μl of 10 μmol/L individual‐specific primer mix, 4.35 μl of ddH_2_O, and 0.2 μl GoTaq^®^ Flexi DNA polymerase. In order to prepare a single master mix per 96‐well plate, the well‐specific barcode sequence mix was pipetted to a fresh plate using a multichannel pipet, then the master mix (without primers and PCR1 product) was added using a stepper, and finally, the cleaned and pooled PCR1 product was transferred to the cocktail using a multichannel pipet resulting in a final reaction volume of 11 μl. PCR2 amplification was as follows: 95°C—2 min, 11 cycles [95°C—45 s, 56°C—45 s, 72°C—45 s], 72°C—5 min, 4°C—∞.

Purification of individually barcoded PCR2 products was performed with Agencourt^®^ Am‐Pure^®^ XP magnetic beads. First, PCR2 products were diluted with ddH_2_O in the 96‐well reaction plate to reach a volume of 25 μl, 17.5 μl of beads were then added to each well, and the plate was briefly vortexed and centrifuged. Following 5 min of incubation, the plate was put on a magnetic rack for 5 min where PCR2 binds to the beads which are immobilized on the magnetic rack. The supernatant was discarded and PCR2 was washed twice with 100 ml freshly prepared 80% ethanol, followed by 5 min of incubation to let evaporate remaining ethanol traces. The purified PCR2 products were then eluted with 20 μl DNase‐free and RNase‐free ddH_2_O and 10 μl of the purified PCR2 was transferred to fresh 96‐well plates.

DNA concentration of PCR2 products was determined using the PicoGreen^®^ assay on a multimode microplate reader (TECAN) according to the manufacturer's protocol. Subsequently, for each individual per 96‐well plate, 50 ng of PCR2 product was pooled into a 1.5‐ml Eppendorf tube, and thus, 16 tubes each containing a 96‐well plate library containing the individually barcoded amplicons of up to 96 individuals were created. A volume of 50 μl from each of the 16 tubes was then transferred into a fresh 1.5‐ml Eppendorf tube and a second round of magnetic bead purification was performed on a magnetic rack compatible with 1.5‐ml Eppendorf tubes. 35 μl of beads was added to each of the 16 reaction tubes, followed by short vortexing and centrifuging. After 5 min of incubation, the tubes were put on the magnetic rack for 5 min and the supernatant was discarded. Two wash steps with 200 ml 80% ethanol were effected, followed by 5 min of incubation. The purified PCR2 plate libraries were eluted with 25 μl DNase‐free and RNase‐free ddH_2_O and 23 μl was transferred to fresh tubes. PCR2 concentrations of the 16 PCR2 plate libraries were measured using the PicoGreen^®^ assay. In a final step, 200 ng of each purified PCR2 plate library for each full 96‐well plate (or proportional lower depending on the number of individuals per plate) was pooled together in a single 1.5‐ml Eppendorf tube. The pooled and purified PCR2 library was paired‐end sequenced on an Illumina^®^ MiSeq‐Chip using a 600 cycle v3 kit according to the manufacturer′s instructions.

### 2.5 Bioinformatics

Sequencing reads were demultiplexed according to the sample‐specific barcodes on the Illumina^®^ MiSeq system into left (5′–3′) and right (3′–5′) fastq files for each of the 1,505 individuals. In a first step, all left and all right fastq files were concatenated into combined left and right files. The program FastQC v. 0.11.5 (Andrews, 2010) revealed fragment length distributions of 35–300 bp for the 11.653.230 paired‐end reads. In a next step, adapter contamination removal and quality and size trimming were done using the program Trimmomatics (Bolger, Lohse, & Usadel, 2014) with the following parameters: “leading” and “trailing” = 20, “sliding window” = 20:20, “minlen” = 200. Subsequently, the cleaned paired‐end reads were merged using the program FLASH (Magoč & Salzberg, 2011) with minimum overlap of 50 bp and maximum overlap of 250 bp. Paired reads were then aligned to the amplicon sequences ±50 bp (VGLL3_323 ± 50 bp, VGLL3_54 ± 50 bp, AKAP11 ± 50 bp, SIX6 ± 50 bp) that were used for the primer design. Sequence reads were mapped to this reference sequence using the bwa mem algorithm from the Burrows‐Wheeler Aligner software package (Li, 2013). The program samtools (Li et al., 2009) was used to convert and sort the sequence alignment files (SAM format) into binary alignment files (BAM format), and to index the BAM files. SNP variants were then called using the samtools mpileup command with parameters ‐uD, ‐E, and ‐f (Li et al., 2009). The program vcftools (Danecek et al., 2011) was used to filter for SNPs that exhibited minor allele frequencies of <1% and minimum mean sequencing depths of 50 over all individuals. For downstream analysis, a data matrix was created containing the genotype information for each SNP and individual along with the classifier information on sex, population, sampling year, sea stage (1SW/MSW), and fork length. Data analysis was conducted in R (R Core Team 2014). To reduce the likelihood of false classification of heterozygous individuals being homozygous at a given SNP, only variant calls with read depths of 5× at the individual level were kept for downstream analysis.

### 2.6 Correlation of SNP variation with sea age

Although more than 1,500 individuals were investigated, the genotype sample sizes for some groups (notably 1SW females and MSW males in some rivers) were small, and for some groups, no data were available (Table 1). This low representation of 1SW females and MSW males in some rivers reflects the demographic reality of those populations (Cauchon, 2015). To infer systematic patterns of SNP genotype variation associated with sea age and sex, we calculated proportions of individuals carrying the common SNP genotype in relation to alternative genotypes. This in turn facilitated across population comparisons. To infer whether the differences in the genotype distributions between 1SW and MSW fish in each sex and population were representative and not artifacts of unequal sample sizes (Table 1), we conducted chi‐square statistics with and without multiple test correction. Chi‐square statistics were also conducted to test for statistically significant differences in the genotype distribution between sexes, that is, within a sea age class.

**Table 1:** Genetic variation at the four candidate loci indicated by the number of individuals bearing a particular genotype. E = Early, L = Late

River	Sex	Sea age	*n* Max	Asn323Lys_*vgll3*_			Met54Thr_*vgll3*_			*SIX6*_TOP_			*SIX6*_EXT_		
				EE	EL	LL	EE	EL	LL	AA	AG	GG	CC	TC	TT
Escoumins	**♀**	1SW	6	0	5	1	0	5	1	0	3	3	3	3	0
Escoumins	**♀**	MSW	209	8	69	132	8	70	129	35	109	59	58	105	40
Escoumins	**♂**	1SW	70	0	18	52	0	18	52	14	38	16	16	39	13
Escoumins	**♂**	MSW	80	1	15	64	1	16	62	22	42	15	15	42	22
			Σ	9	107	249	9	109	244	71	192	93	92	189	75
Malbaie	**♀**	1SW	6	0	4	2	0	4	2	1	3	0	0	3	1
Malbaie	**♀**	MSW	188	2	71	115	5	68	113	41	84	50	49	85	41
Malbaie	**♂**	1SW	135	6	37	92	6	38	91	28	66	36	35	67	28
Malbaie	**♂**	MSW	50	2	10	38	2	11	37	19	20	10	10	20	19
			Σ	10	122	247	13	121	243	89	173	96	94	175	89
Trinité	**♀**	1SW	30	1	13	16	1	12	17	3	10	13	12	11	3
Trinité	**♀**	MSW	205	0	30	175	0	30	172	16	79	85	79	90	15
Trinité	**♂**	1SW	228	2	27	199	2	27	197	20	82	100	91	89	22
Trinité	**♂**	MSW	16	0	0	16	0	0	16	2	6	6	6	6	2
			Σ	3	70	406	3	69	402	41	177	204	188	196	42
Vieux‐Fort	**♀**	1SW	94	0	8	86	0	8	86	0	8	76	30	42	12
Vieux‐Fort	**♀**	MSW	71	0	5	66	0	4	66	0	3	56	22	30	8
Vieux‐Fort	**♂**	1SW	64	0	5	59	0	5	59	0	6	51	19	30	9
Vieux‐Fort	**♂**	MSW	10	0	2	8	0	2	8	0	1	7	1	4	2
			Σ	0	20	219	0	19	219	0	18	190	72	106	31
			**Σ genotype**	22	319	1121	25	318	1108	201	560	583	446	666	237
			**Σ locus**	1462			1451			1344			1349		

A complementary binary logistic regression considering the factors sex, year, and river as explanatory variables targeted the predictability of sea age from SNP genotypes. Logistic regression is a special case of generalized linear mixed‐effects model with a binomial error distribution (1 = “MSW,” 0 = “1SW”). A model selection process based on the Akaike information criterion (AIC) was first conducted to identify the model that best fitted our data (Burnham & Anderson, 2004) (Table S3). The *glmer* function package “*lme4*” (Bates, 2005) was used to fit the models. The best model was then fully explored, and the estimated posterior distribution of the model parameters was assessed using the *sim* function (package “*arm*” (Gelman & Hill, 2007)) based on simulation of 5,000 values of the posterior model parameter distribution. Inference was drawn based on the 95% credible interval (CrI), which is the Bayesian analog to confidence interval. Conventionally, if zero is not included in the Bayesian 95% CrI, an effect is considered to be “clear.” Modeling results were interpreted as the probability for a given genotype at a given SNP of being MSW under the null hypothesis that MSW and 1SW are equally likely. An additional logistic regression model was designed to specifically tested whether sex could be predicted from SNP genotypes (model formula: sex ~ SNP‐1 + (1|river) + (1|year) under the null hypothesis that a given individual is equally likely to be of male or female sex.

## 3 RESULTS

### 3.1 Bioinformatics

Mean coverage per amplicon and individual was around 1000× for amplicons VGLL3_54, VGLL3_323, and AKAP11. Coverage of the SIX6 amplicon, however, was approximately 100× only. Four SNPs passed the filtering criteria, three of which were identified as the target SNPs (Asn323Lys_*vgll3*_, Met54Thr_*vgll3*_, and *SIX6*_TOP_). An additional SNP was identified in the SIX6 amplicon and was termed *SIX6*_EXT_. Although the fourth target (Val214Met_*akap11*_) was present in the data set, it has been filtered out as the minor allele frequency of this SNP was <1%. For individual downstream analyses, genotype calls of 1,451, 1,462, 1,344, and 1,349 individuals were available for SNPs Met54Thr_*vgll3*,_ Asn323Lys_*vgll3*_, *SIX6*_TOP_, and *SIX6*_EXT_ (Table 1). Although Met54Thr_*vgll3*_ and Asn323Lys_*vgll3*_ are located on two different amplicons, their genotype distributions were tightly correlated (r correlation coefficient of Met54Thr_*vgll3*_ and Asn323Lys_*vgll3*_ = 0.96 at *p *< .0001; Table 1), as was expected due to their physical proximity and thus linkage and cosegregation (Ayllon et al., 2015; Barson et al., 2015).

### 3.2 Association of genotype proportions with sea age

**Figure 2:**
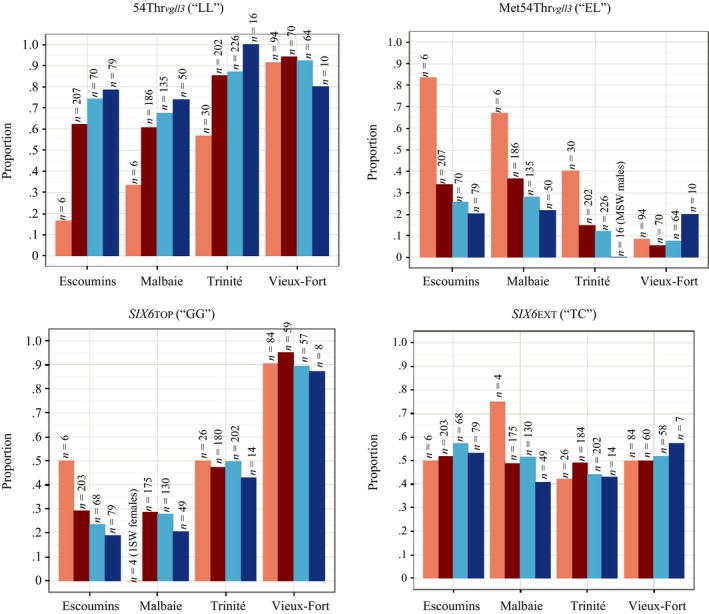
Candidate SNP genotype frequencies across rivers. 1SW females, MSW females, 1SW males, and MSW males are coded in light red, dark red, light blue, and dark blue. Top left: The MSW component of each sex exhibited higher proportions of 54Thr_*vgll3*_ (“LL”) compared to 1SW fish in three of four populations. Top right: The prevalence of being heterozygous (“EL”) for Met54Thr_*vgll3*_ was higher in females except for Vieux‐Fort River. Bottom: No systematic pattern was found for any *six6* SNP

54Thr_*vgll3*_ (“CC”) and 323Lys_*vgll3*_ (“GG” were most abundant in all populations and more than 75% of the individuals carried these genotypes. Genotype proportions were calculated for each sex and sea age category to facilitate comparisons within and across populations. We found that in three of four rivers, the MSW population component exhibited higher proportions of variants 54Thr_*vgll3*_ (“CC”), hereinafter 54Thr_*vgll3*_ (“LL”), and 323Lys_*vgll3*_ (“GG”), hereinafter 323Lys_*vgll3*_ (“LL”), compared to 1SW groups, with females exhibiting more pronounced differences than males (Figure 2, Table 2). For example, in the Escoumins River, only 17% of 1SW females exhibited 54Thr_*vgll3*_ (“LL”), whereas 62% of the MSW females carried this genotype. A similar trend can be observed in the populations Malbaie (33% in 1SW females vs. 61% in MSW females) and Trinité (57% in 1SW females vs. 85% in MSW females) (Figure 2). However, the Vieux‐Fort River population with a very high proportion of ca. 90% 1SW fish in both sexes (Cauchon, 2015) did not conform to the pattern found in the other three populations (Figure 2). We did not detect any systematic pattern in the distribution of the *six6* SNPs (Table 2).

**Table 2:** Chi‐square summary statistics comparing the genotype distributions between 1SW and MSW fish, that is, within sexes (left column) and between sexes, that is, within sea age categories (right column). Statistical inference is given without (*p*) and with (*p* corrected) Bonferroni correction. Sample sizes are inferable from Table 1. *P*‐values set in boldface indicate statistical significance

SNP	River	Sex	χ^2^	*df*	*p*	*p* corrected	SNP	river	sea age	χ^2^	*df*	*p*	*p* corrected
Asn323Lys_*vgll3*_	Escoumins	**♂**	1.86	2	.40	.78	Asn323Lys_*vgll3*_	Escoumins	MSW	7.70	2	**.021**	.117
Asn323Lys_*vgll3*_	Escoumins	**♀**	6.56	2	**.04**	.29	Asn323Lys_*vgll3*_	Escoumins	1SW	6.18	1	**.013**	.085
Asn323Lys_*vgll3*_	Trinité	**♂**	2.31	2	.32	.78	Asn323Lys_*vgll3*_	Trinité	MSW	1.61	1	.205	.398
Asn323Lys_*vgll3*_	Trinité	**♀**	21.92	2	**<.0001**	**<.001**	Asn323Lys_*vgll3*_	Trinité	1SW	22.00	2	**<.0001**	**<.001**
Asn323Lys_*vgll3*_	Malbaie	**♂**	1.12	2	.57	.78	Asn323Lys_*vgll3*_	Malbaie	MSW	7.04	2	**.030**	.127
Asn323Lys_*vgll3*_	Malbaie	**♀**	2.07	2	.36	.78	Asn323Lys_*vgll3*_	Malbaie	1SW	4.36	2	.113	.311
Asn323Lys_*vgll3*_	Vieux‐Fort	**♂**	0.41	1	.52	.78	Asn323Lys_*vgll3*_	Vieux‐Fort	MSW	0.58	1	.445	.647
Asn323Lys_*vgll3*_	Vieux‐Fort	**♀**	0.00	1	.96	1.00	Asn323Lys_*vgll3*_	Vieux‐Fort	1SW	0.00	1	1.000	1.000
Met54Thr_*vgll3*_	Escoumins	**♂**	1.46	2	.48	.78	Met54Thr_*vgll3*_	Escoumins	MSW	6.96	2	**.031**	.127
Met54Thr_*vgll3*_	Escoumins	**♀**	6.28	2	**.04**	.29	Met54Thr_*vgll3*_	Escoumins	1SW	6.18	1	**.013**	.085
Met54Thr_*vgll3*_	Trinité	**♂**	2.33	2	.31	.78	Met54Thr_*vgll3*_	Trinité	MSW	1.65	1	.199	.398
Met54Thr_*vgll3*_	Trinité	**♀**	18.46	2	**<.0001**	**<.01**	Met54Thr_*vgll3*_	Trinité	1SW	17.98	2	**<.001**	**<.01**
Met54Thr_*vgll3*_	Malbaie	**♂**	0.77	2	.68	.90	Met54Thr_*vgll3*_	Malbaie	MSW	3.81	2	.149	.336
Met54Thr_*vgll3*_	Malbaie	**♀**	2.30	2	.32	.78	Met54Thr_*vgll3*_	Malbaie	1SW	4.14	2	.126	.320
Met54Thr_*vgll3*_	Vieux‐Fort	**♂**	0.41	1	.52	.78	Met54Thr_*vgll3*_	Vieux‐Fort	MSW	0.93	1	.336	.554
Met54Thr_*vgll3*_	Vieux‐Fort	**♀**	0.14	1	.71	.90	Met54Thr_*vgll3*_	Vieux‐Fort	1SW	0.00	1	1.000	1.000
*SIX6*_TOP_	Escoumins	**♂**	1.19	2	.55	.78	*SIX6*_TOP_	Escoumins	MSW	5.37	2	.068	.250
*SIX6*_TOP_	Escoumins	**♀**	1.91	2	.38	.78	*SIX6*_TOP_	Escoumins	1SW	2.77	2	.250	.449
*SIX6*_TOP_	Trinité	**♂**	0.38	2	.83	.95	*SIX6*_TOP_	Trinité	MSW	0.46	2	.793	1.000
*SIX6*_TOP_	Trinité	**♀**	0.36	2	.83	.95	*SIX6*_TOP_	Trinité	1SW	0.09	2	.957	1.000
*SIX6*_TOP_	Malbaie	**♂**	5.50	2	.06	.31	*SIX6*_TOP_	Malbaie	MSW	4.74	2	.093	.287
*SIX6*_TOP_	Malbaie	**♀**	1.73	2	.42	.78	*SIX6*_TOP_	Malbaie	1SW	1.57	2	.456	.647
*SIX6*_TOP_	Vieux‐Fort	**♂**	0.00	1	1.00	1.00	*SIX6*_TOP_	Vieux‐Fort	MSW	0.00	1	.972	1.000
*SIX6*_TOP_	Vieux‐Fort	**♀**	0.44	1	.51	.78	*SIX6*_TOP_	Vieux‐Fort	1SW	0.00	1	1.000	1.000
*SIX6*_EXT_	Escoumins	**♂**	1.64	2	.44	.78	*SIX6*_EXT_	Escoumins	MSW	3.76	2	.153	.336
*SIX6*_EXT_	Escoumins	**♀**	2.10	2	.35	.78	*SIX6*_EXT_	Escoumins	1SW	2.70	2	.259	.449
*SIX6*_EXT_	Trinité	**♂**	0.15	2	.93	1.00	*SIX6*_EXT_	Trinité	MSW	0.67	2	.716	.945
*SIX6*_EXT_	Trinité	**♀**	0.57	2	.75	.92	*SIX6*_EXT_	Trinité	1SW	0.03	2	.985	1.000
*SIX6*_EXT_	Malbaie	**♂**	5.47	2	.06	.31	*SIX6*_EXT_	Malbaie	MSW	4.69	2	.096	.287
*SIX6*_EXT_	Malbaie	**♀**	1.68	2	.43	.78	*SIX6*_EXT_	Malbaie	1SW	1.51	2	.471	.647
*SIX6*_EXT_	Vieux‐Fort	**♂**	1.36	2	.51	.78	*SIX6*_EXT_	Vieux‐Fort	MSW	1.95	2	.377	.592
*SIX6*_EXT_	Vieux‐Fort	**♀**	0.03	2	.98	1.00	*SIX6*_EXT_	Vieux‐Fort	1SW	0.14	2	.931	1.000

The difference in the *vgll3* genotype distributions between 1SW and MSW fish in females from the Trinité River was statistically highly significant at *p *< .001 for Asn323Lys_*vgll3*_ and *p *< .01 for Met54Thr_*vgll3*_ (Table 2), thus supporting the hypothesis of genotype–phenotype associations related to *vgll3*. We also confirmed statistically significant differences in the *vgll3* genotype distributions between males and females in 1SW fish from Trinité River at *p *< .001 for Asn323Lys_*vgll3*_ and *p *< .01 for Met54Thr_*vgll3*_ (Table 2). Although three of four populations exhibited a similar trend in the distribution of *vgll3* genotypes (Figure 2), all other comparisons resulted at most in marginally nonsignificant differences (.05 < *p *< .1) in the genotype distributions of the 1SW or MSW group or between the sexes of a given sea age class (Table 2). Given the clear trend throughout three populations, we believe that the nonsignificance of *vgll3* genotype comparisons is possibly partly due to unbalanced sample sizes (Table 1). We did not detect any significant differences in the distributions of the *six6* genotypes between 1SW/MSW or between sexes.

### 3.3 Complementary analysis: Predicting sea age from SNP genotypes

The logistic regression model with the three‐way interaction of SNP genotypes, sex, and population (river) was identified as the best fitting model (Table S3). Although the late‐maturing *vgll3* genotypes 54Thr_*vgll3*_ (“LL”) and 323Lys_*vgll3*_ (“LL”) were most abundant in all four populations (Table 1), the logistic regression analysis of *vgll3* SNP genotypes revealed increased probabilities of belonging to the MSW group for genotypes 54Thr_*vgll3*_ (“LL”) and 323Lys_*vgll3*_ (“LL”) compared to alternative genotypes (“EL,” “EE”), depending on population and sex (Table S4).

### 3.4 Genotype frequencies and sex

Genotype frequencies varied between sexes in *vgll3*, that is, individuals carrying the heterozygous *vgll3* SNP genotypes were more likely to be female (Met54Thr_*vgll3*_ (“EL”) = 66% probability of being female, 95% CrI = 55%–76%, *n *=* *1451; Asn323Lys_*vgll3*_ (“EL”) = 67% probability of being female, 95% CrI = 56%–77%, *n *=* *1462). No systematic effect of sex on any other genotype was detected.

## 4 DISCUSSION

### 4.1 Variation at a major sea age locus in North American Atlantic Salmon

In our data set of 1,505 Atlantic Salmon from four North American populations, we confirmed the presence of two nonsynonymous mutations within the *vgll3* gene that have been documented in European populations. Moreover, these SNPs seem to associate with age at maturity to a certain extent that is the association varied with sex and population in our study system. In one population (Trinité), the SNP genotypes 54Thr_*vgll3*_ (“LL”) and 323Lys_*vgll3*_ (“LL”) occur in significantly higher proportions in late‐maturing females compared to early‐maturing fish and a similar trend can be observed in two further populations (Escoumins and Malbaie). In contrast, although males from those populations (Escoumins, Malbaie, and Trinité) exhibited the same trend in the *vgll3* SNP genotype distributions, this pattern never resulted in statistical significance, suggesting that the genotype–phenotype association may be more pronounced in females than in males among those North American populations. Previous work showed that those genotypes are associated with higher maturation ages in European populations (Ayllon et al., 2015), lending support to the hypothesis that these SNPs correlate with sea age throughout the native range of this species. Moreover, the linkage disequilibrium between Met54Thr_*vgll3*_ and Asn323Lys_*vgll3*_ was extremely strong, which raises the possibility that both mutations may act in concert to influence sea age (Ayllon et al., 2015; de Juan, Pazos, & Valencia, 2013).

Interestingly, the late‐maturing *vgll3* genotypes 54Thr_*vgll3*_ (“LL”) and 323Lys_*vgll3*_ (“LL”) were most abundant in all four populations (between 65% and 92%; Table 1). Moreover, compared to the European study (Barson et al., 2015), the overall prevalence of 1SW females was very low and the fact that >3SW fish are generally rare in North American populations (Cauchon, 2015) implies that the MSW components of our populations likely contained no or very few >3SW fish. This may suggest that the antagonistic selection pressures between European and North American populations could differ. Furthermore, our sampling did not allow to test for the sex‐dependent prevalence of alternative homozygous genotypes in younger and older >3SW fish that supposedly contributes to the resolution of sexual conflict in the species (Barson et al., 2015). Therefore, future studies targeting North American *vgll3* variation should ideally include populations with both high proportions of 1SW females and >3SW individuals of both sexes. These samples, however, are quite rare in the rivers studied here (Cauchon, 2015) and were not available for our study. Future studies would also benefit from comparisons of *vgll3* genotype distributions before and after the marine migration in order to test for differential mortality associated with *vgll3* genotypes.

### 4.2 Parallelism and population‐specific effects

Although the extent of genetic divergence and life‐history traits varied among the populations we studied, we observed a similar distribution of *vgll3* genotypes across sea age classes and sexes in three of four investigated populations and differences between sea age classes in *vgll3* genotype distributions were stronger in females than in males. However, the fourth population from the Vieux‐Fort River clearly stands out. The Vieux‐Fort River has a very untypical life‐history structure compared to other populations from Québec with more than 90% 1SW fish in both sexes (Cauchon, 2015). These characteristic differences in the population structure might reflect substantially varying selection pressures across rivers. It is also conceivable that a large proportion of the variation in sea age is brought about by other, so far undetermined genes or environmental factors that contribute to the prevalence of the 1SW trait.

### 4.3 SNP genotypes and sex

Genotype frequencies varied between sexes, that is, heterozygous individuals at both investigated nonsynonymous *vgll3*‐SNPs had an approximately 2:1 probability of being female and not male. The 2:1 probability of being female when heterozygous for *vgll3* possibly reflects the influence of the late‐maturing allele on delaying sea age (Barson et al., 2015) compared to an individual being homozygous for the early allele. The result can also be interpreted in the context of the substantially different reproductive strategies across sexes with the females maturing later and at larger sizes on average relative to males. Thus, carrying a late‐maturing allele could hypothetically be more necessary for female survival and reproductive success than it is for males. A direct link of *vgll3* with sex determination in Atlantic Salmon could be an alternative explanation which cannot be addressed in this study. Clearly, this deserves further investigation in North American populations.

### 4.4 Beyond the species border—transferability to other salmonids?

Met54Thr_*vgll3*_ and Asn323Lys_*vgll3*_ are also present across Brown Trout (*Salmo trutta*), Rainbow Trout (*Oncorhynchus mykiss*), and Arctic Char (*Salvelinus alpinus*) (Ayllon et al., 2015), thus lending support to the idea that *vgll3* influences sea age in multiple species. Ayllon et al. (2015) reported that 3SW individuals of these species all possess the amino acid variants that associate with later maturation in our sample, that is, 54Thr_*vgll3*_ (“LL”) and 323Lys_*vgll3*_ (“LL”) (Ayllon et al., 2015). An allelic discrimination assay on a small sample of five individuals of a landlocked Swedish Atlantic Salmon population further confirmed the 3SW variants of *vgll3* for all five individuals, and it was concluded that European Atlantic Salmon 3SW variants (54Thr_*vgll3*_ and 323Lys_*vgll3*_) are ancestral whereas the 1SW variants of 54Met_*vgll3*_ and 323Asn_*vgll3*_) are derived (Ayllon et al., 2015). While the presence of Met54Thr_*vgll3*_ and Asn323Lys_*vgll3*_ is unchallenged, it is noteworthy that in our data set, between 65% and 92% of the individuals within a population carried the “ancestral” 54Thr_*vgll3*_ (“LL”) and 323Lys_*vgll3*_ (“LL”) variants (Table 1). In light of the low prevalence of late‐maturing fish in North American Atlantic Salmon populations this suggests that different selection pressures might be at work at either side of the Atlantic Ocean.

## CONFLICT OF INTEREST

None declared.

## AUTHOR CONTRIBUTIONS

LB and HK conceived the study. HK, GC, CH, and LB implemented the methodology. HK and DBD conducted the laboratory work. EN and HK developed the bioinformatics pipeline. HK analyzed the data with EN. HK, LB, and EN interpreted the data. HK drafted the manuscript. All authors read and approved the final manuscript.
