# Supplementary material for: Characterization of natural variation in North American Atlantic Salmon populations (Salmonidae: Salmo salar) at a locus with a major effect on sea age
Source: Ecol Evol. 2017 Jun 17;7(15):5797–807. doi: 10.1002/ece3.3132 (PMC5550958; doi:10.1002/ece3.3132)
Supplement: Supplementary file 1 [file ECE3-7-5797-s001.pdf]

Supplementary Material: "Characterization of natural variation in North American Atlantic Salmon populations (Salmonidae*: Salmo salar*) at a locus with a major effect on sea age"

Supplementary Table 1. Sample sizes for resequencing assay.

**river**	**year**	***n* 1SW**	***n* MSW**
Malbaie	2003	50	77
Malbaie	2004	50	100
Malbaie	2005	50	68
		**Σ 150**	**Σ 245**
Escoumins	2009	50	100
Escoumins	2010	0	96
Escoumins	2011	30	100
		**Σ 80**	**Σ 296**
Trinité	2010	50	30
Trinité	2011	50	42
Trinité	2012	50	61
Trinité	2013	32	36
Trinité	2014	29	16
Trinité	2015	50	43
		**Σ 261**	**Σ 228**
Vieux-Fort	2011	43	16
Vieux-Fort	2012	35	3
Vieux-Fort	2013	23	11
Vieux-Fort	2014	40	10
Vieux-Fort	2015	21	43
		**Σ 162**	**Σ 83**

$\mathbf{\Sigma_{1SW} = 653}$ $\mathbf{\Sigma_{MSW} = 852}$

$\mathbf{\Sigma_{Total} = 1505}$

Supplementary Table 2. PCR primer sequences to target the non-synonymous SNPs in *vgll3* and *akap11*, as well as for SNPs $SIX6_{\mathrm{TOP}}$ (Barson *et al.* 2015) and $SIX6_{\mathrm{EXT}}$.

name	target	product size	forward primer	reverse primer
VGLL3_323	Asn323Lys$_{vgll3}$	406 bp	CTCCAACCTCTCTCCCTGAC	CTCCAGGTCAGAGGCTTACC
VGLL3_54	Met54Thr$_{vgll3}$	444 bp	TGGCTGAATCTGTGGCATAC	GTGTGCTCCTACCTTTCCAC
AKAP11	Val214Met$_{akap11}$	433 bp	AGTCTCGGTTACCACTGCTC	CAGAGTCAGAGTCCTCCACC
SIX6	$SIX6_{\mathrm{TOP}}$, $SIX6_{\mathrm{EXT}}$	433 bp	ATATCAAAGGGAACGAACGGT	ACGGGGTAAGTAATCGCTGT

Supplementary Table 3. Model selection based on AIC comparisons. Model 1 with the three-way interaction of SNP genotypes, sex and river was identified as the best model for all four candidate SNPs. For all four SNP candidates, the model 1 fitted the data better than the null model (sea age ~ sex:river-1 + (1|year); df = 9, AIC = 1130.218).

		**Asn323Lys*vgll3***			**Met54Thr*vgll3***			***SIX6*TOP**			***SIX6*EXT**		
**model**	**model description**	**df**	**AIC**	**ΔAIC**	**df**	**AIC**	**ΔAIC**	**df**	**AIC**	**ΔAIC**	**df**	**AIC**	**ΔAIC**
**1**	**sea age ~ SNP:sex:river-1 + (1|year)**	**23**	**1111.2**	**0.0**	**23**	**1111.5**	**0.0**	**23**	**1029.4**	**0.0**	**25**	**1030.4**	**0.0**
2	sea age ~ SNP-1 + sex + SNP:sex + (1|river) + (1|year)	8	1156.1	44.9	8	1154.9	43.3	8	1057.8	28.4	8	1049.1	18.7
3	sea age ~ SNP:sex-1 + (1|river) + (1|year)	8	1156.1	44.9	8	1154.9	43.3	8	1057.8	28.4	8	1049.1	18.7
4	sea age ~ SNP-1 + (sex) + (1|river) + (1|year)	6	1154.7	43.5	6	1153.6	42.1	6	1054.2	24.8	6	1049.0	18.6
5	sea age ~ SNP-1 + (1|sex) + (1|river) + (1|year)	6	1163.2	52.0	6	1162.1	50.6	6	1062.6	33.2	6	1057.3	27.0
6	sea age ~ SNP-1 + (1|sex) + (1|river)	5	1175.2	64.0	5	1172.6	61.0	5	1072.9	43.5	5	1067.6	37.2
7	sea age ~ SNP-1 + (1|sex)	4	1470.6	359.5	4	1462.7	351.2	4	1279.6	250.2	4	1338.2	307.8

Supplementary Table 4. Percentage probability of being MSW and not 1SW for candidate SNP genotypes across sexes and populations (mean and 95 % Bayesian credible interval in parentheses, null hypothesis = 50 %). Results refer to the best model investigating the three-way interaction of SNP genotypes, sex and river (model 1 in Supplementary Table 3). Non-significant comparisons are marked "ns" and "NA" indicates lacking data.

		**Asn323Lys*vgll3***			**Met54Thr*vgll3***			***SIX6*TOP**			***SIX6*EXT**		
		**EE**	**EL**	**LL**	**EE**	**EL**	**LL**	**AA**	**AG**	**GG**	**CC**	**TC**	**TT**
Escoumins	♀	ns	95 (87-98)	99 (96-100)	ns	95 (87-98)	99 (95-100)	ns	98 (93-99)	97 (89-99)	97 (89-99)	98 (93-99)	ns
	♂	ns	ns	64 (50-76)	ns	ns	ns	70 (52-84)	ns	ns	ns	ns	72 (53-86)
Malbaie	♀	ns	95 (85-98)	98 (93-100)	ns	95 (85-98)	98 (93-100)	98 (85-100)	97 (89-99)	ns	ns	97 (89-99)	98 (84-100)
	♂	ns	21 (10-39)	29 (18-43)	ns	22 (11-39)	29 (18-42)	ns	23 (13-37)	21 (10-38)	22 (11-40)	23 (13-38)	ns
Trinité	♀	ns	ns	91 (85-95)	ns	31 (18-50)	90 (84-95)	82 (56-95)	88 (77-94)	86 (75-92)	86 (74-92)	88 (78-94)	82 (55-94)
	♂	ns	ns	6 (3-10)	ns	ns	6 (3-11)	8 (2-28)	6 (2-13)	5 (2-11)	5 (2-11)	5 (2-11)	7 (2-25)
Vieux-Fort	♀	NA	ns	ns	NA	ns	ns	NA	ns	ns	ns	ns	ns
	♂	NA	ns	11 (5-23)	NA	ns	11 (6-23)	NA	ns	11 (5-22)	5 (1-29)	11 (4-27)	14 (3-45)

Supplementary Figure 1. Definition of sea age categories based on fork length. Specimen illustration by Knepp, Timothy - U.S. Fish & Wildlife Service. Licensed under public domain via Wikimedia Commons – http://commons.wikimedia.org.

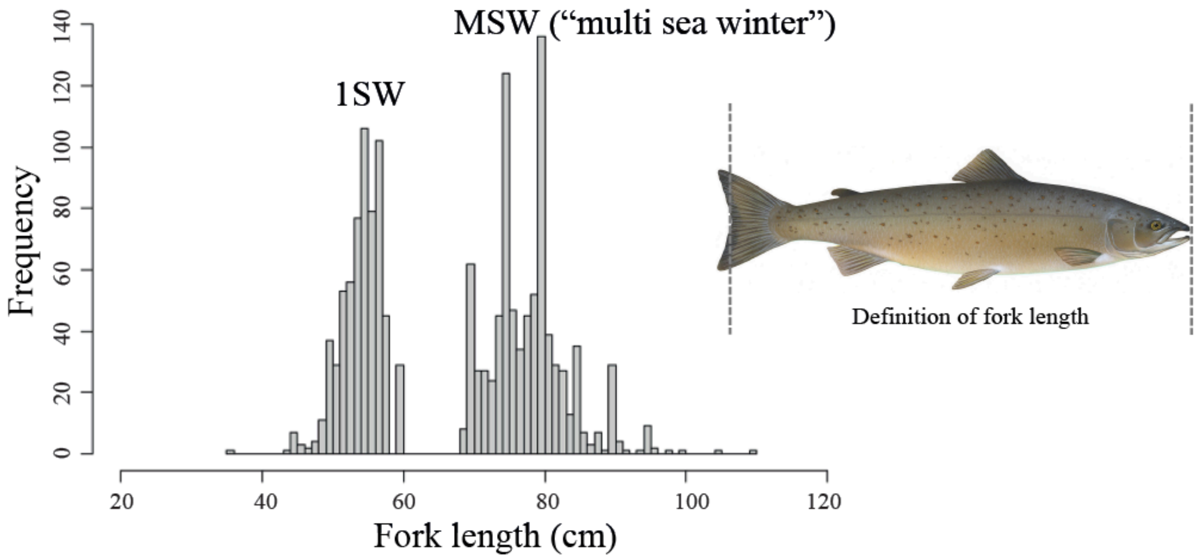

Supplementary Figure 2A-D. Genotype distributions of candidate SNPs for sea age in four salmon rivers from Québec, Canada. Data are displayed in two ways, the observed data (Table 1) and adjusted for unequal sample sizes of 1SW and MSW fish to anticipate a distribution what the genotype distribution could be if equal amounts of 1SW and MSW fish were sampled within each sex. Specimen illustrations by Knepp, Timothy - U.S. Fish & Wildlife Service. Licensed under public domain via Wikimedia Commons – http://commons.wikimedia.org.

A)

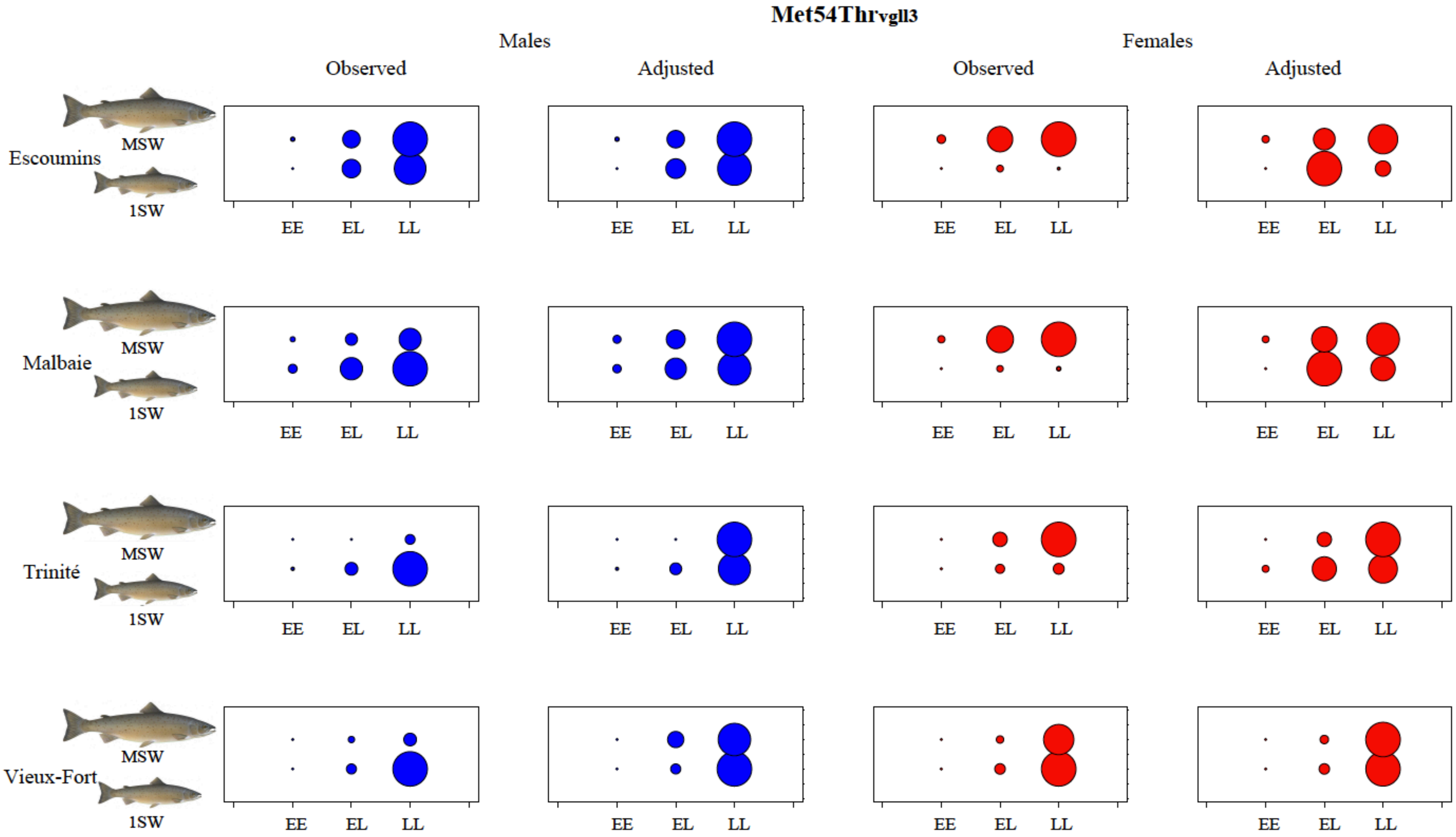
Met54Thrvgll3
Males
Females
Observed
Adjusted
Observed
Adjusted
Escoumins
Malbaie
Trinité
Vieux-Fort
MSW
1SW
EE
EL
LL

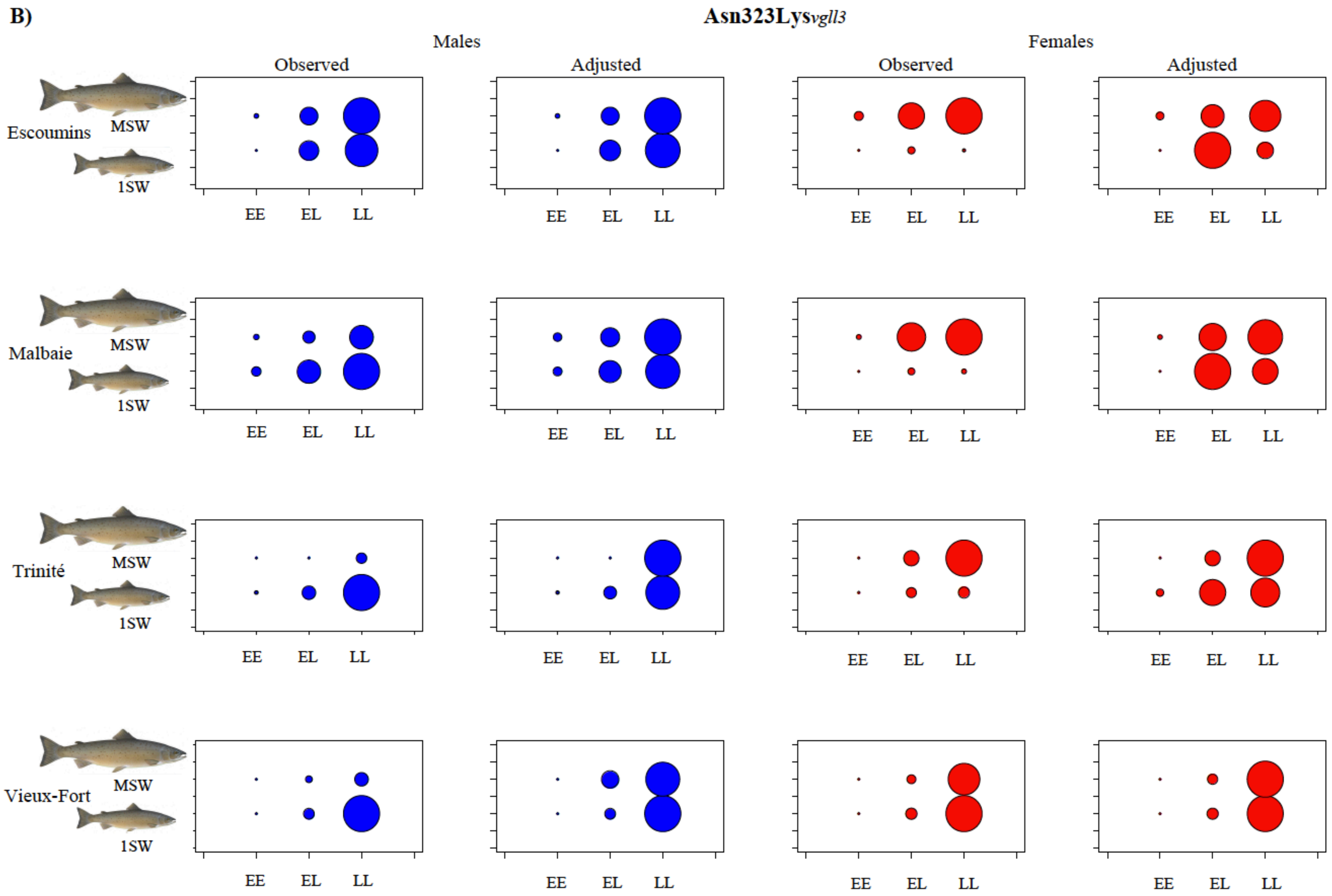
B)
Asn323Lys*vgll3*
Males
Females
Observed
Adjusted
Observed
Adjusted
Escoumins
MSW
1SW
Malbaie
MSW
1SW
Trinité
MSW
1SW
Vieux-Fort
MSW
1SW
EE
EL
LL

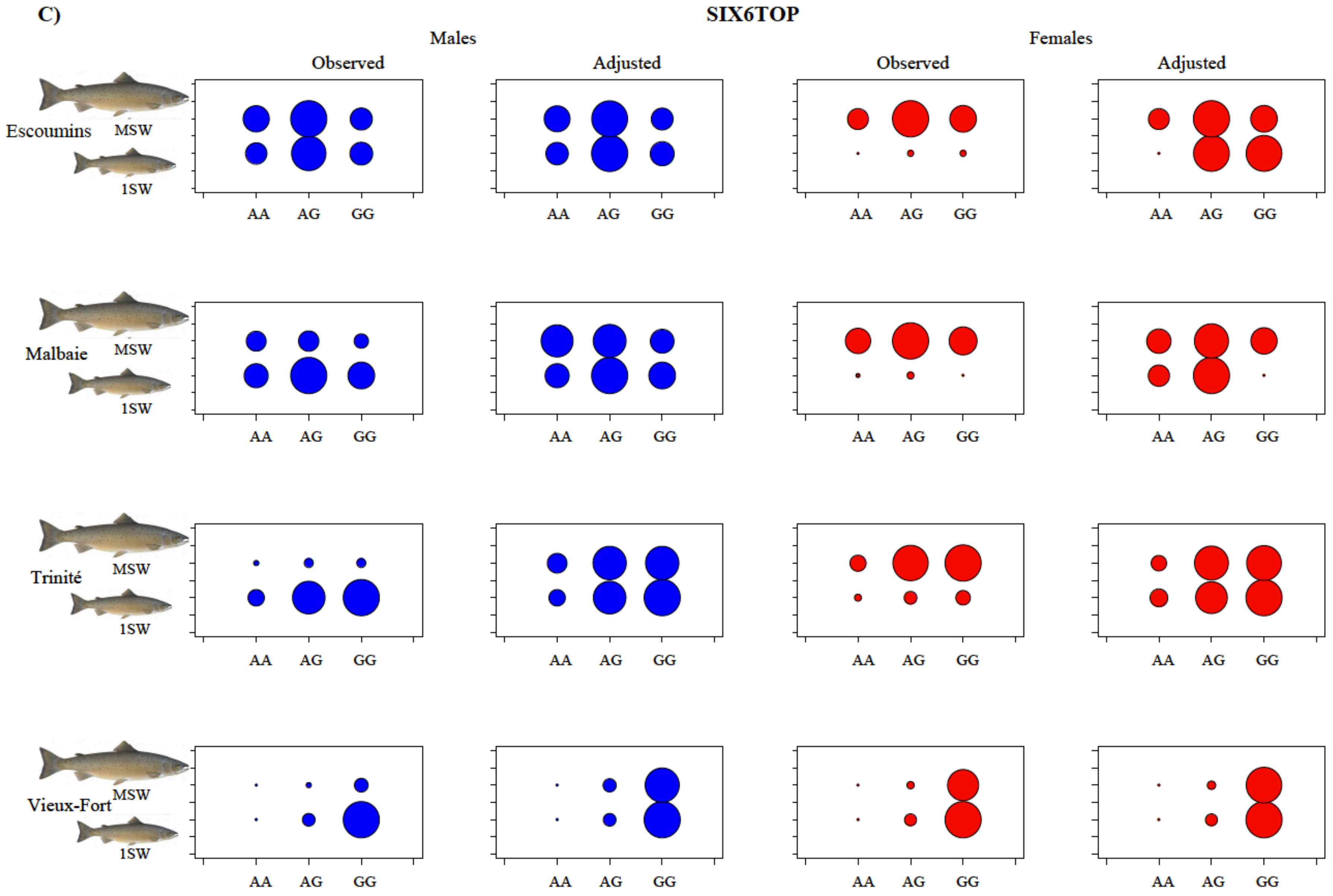
C)
SIX6TOP
Males
Females
Observed
Adjusted
Observed
Adjusted
Escoumins
Malbaie
Trinité
Vieux-Fort
MSW
1SW
AA
AG
GG

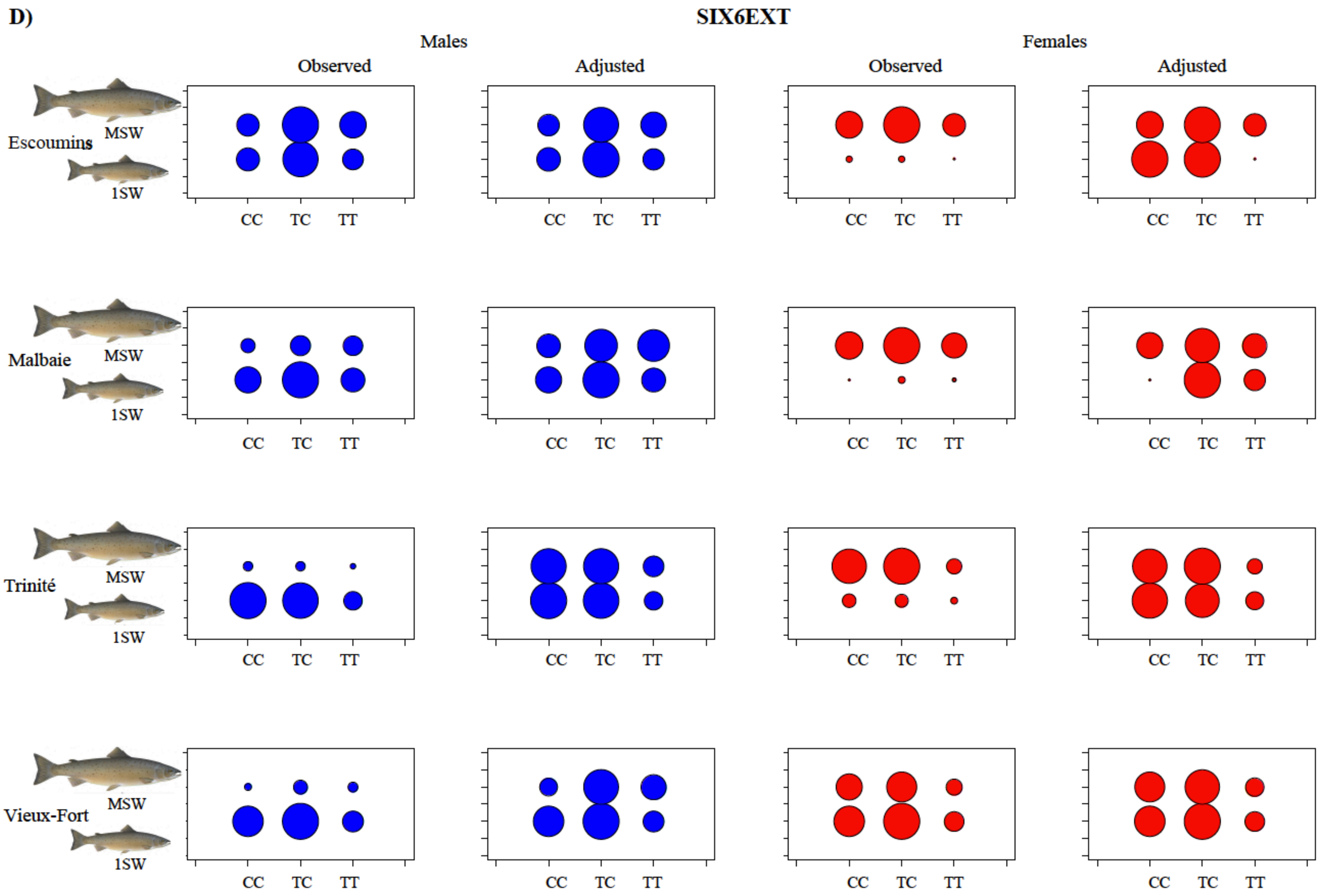
D)
SIX6EXT
Males
Females
Observed
Adjusted
Observed
Adjusted
Escoumins
Malbaie
Trinité
Vieux-Fort
MSW
1SW
CC
TC
TT
